# Molecular Identification of *Borrelia* spp. from Ticks in Pastures Nearby Livestock Farms in Korea

**DOI:** 10.3390/insects12111011

**Published:** 2021-11-09

**Authors:** Haeseung Lee, Seung-Hun Lee, SungShik Shin, Dongmi Kwak

**Affiliations:** 1 College of Veterinary Medicine, Kyungpook National University, 80 Daehak-ro, Daegu 41566, Korea; lhs1457@knu.ac.kr; 2 College of Veterinary Medicine, Chungbuk National University, 1 Chungdae-ro, Cheongju 28644, Korea; dvmshlee@chungbuk.ac.kr; 3 College of Veterinary Medicine, Chonnam National University, 77 Yongbong-ro, Gwangju 61186, Korea; sungshik@jnu.ac.kr; 4 Cardiovascular Research Institute, Kyungpook National University, 680 Gukchaebosang-ro, Daegu 41944, Korea

**Keywords:** *Borrelia*, Korea, phylogeny, tick, tick-borne pathogen

## Abstract

**Simple Summary:**

Lyme borreliosis is caused by a spirochete from the *Borrelia burgdorferi* sensu lato group. *Borrelia afzelii* and *B. garinii* are known to be pathogenic to humans. The main vector for Lyme borreliosis is the *Ixodes* tick. In this study, *Borrelia* infection was confirmed in *Ixodes,* *Haemaphysalis*, and *Amblyomma* ticks. To our knowledge *Borrelia* infection was first confirmed in *Amblyomma testudinarium* in Korea. Based on phylogenetic analysis, all sequences were aligned with *B. afzelii* isolates and showed a close relationship with high identity. Considering that *B. afzelii* causes infectious zoonotic diseases, continuous monitoring and attention are still required (although a low prevalence was recorded in this study).

**Abstract:**

Ticks are vectors that spread pathogenic bacteria, viruses, and protozoa. As the number of ticks increases due to climate change, the importance of the study of tick-borne pathogens has also increased. This study was conducted to investigate the distribution of the major tick species causing Lyme borreliosis and regional differences in the prevalence of *Borrelia* spp. by tick species. *Borrelia* infection was confirmed not only in *Ixodes* ticks, which are the major vectors of *Borrelia* spp., but also in *Haemaphysalis* and *Amblyomma* ticks. PCR targeting the 5S-23S rRNA intergenic spacer region (rrf-rrl) was performed to confirm *Borrelia* positivity. A total of 6102 ticks (736 pools) were tested, and the proportion was *Haemaphysalis longicornis* nymphs and adults at 69.2%, *Haemaphysalis flava* nymphs and adults at 13.9%, *Haemaphysalis* spp. larva at 14.3%, *Ixodes nipponensis* at 0.8%, and *Amblyomma testudinarium* at 1.9%. *Ixodes nipponensis* showed the highest minimum infection rate (MIR: 34.00; 17 pools/50 ticks) for *Borrelia* spp., followed by *A. testudinarium* (MIR: 0.88), and *H. longicornis* (MIR: 0.05). In particular, to our knowledge *Borrelia* infection was first confirmed in *A. testudinarium* in Korea. As a result of phylogenetic analysis, all sequences were grouped with *Borrelia* *afzelii* isolates and showed a close relationship with high identity. Considering that *B. afzelii* causes infectious zoonotic diseases, continuous monitoring and attention are needed, although it has a low prevalence in this study.

## 1. Introduction

Ticks are ectoparasites that feed on humans as well as wild and domestic animals. Ticks are also a vector for spreading viruses and protozoa, as well as bacteria, e.g., *Borrelia*, *Coxiella*, or Rickettsiales [1,2]. Lyme borreliosis is also a tick-borne zoonotic disease. The disease is caused by the spirochete (causative agent) and the most common mode of transmission is via tick bite [3,4]. Although the taxonomy of the Borreliaceae family (genus *Borrelia*) has recently been revised into two genera, *Borrelia* (causing relapsing fever) and *Borreliella* (causing Lyme disease), this change has been challenged [5]. The *Borrelia burgdorferi* s. l. genospecies currently consists of 20 accepted and three proposed genospecies [6], and seven species (*Borrelia afzelii*, *B. garinii*, *B. japonica*, *B. tanukii*, *B. turdi, B. sinica,* and *B. valaisiana*) have been identified in Asia. Of these, *B. afzelii* and *B. garinii* are known to be pathogenic to humans. [4]. The main vectors of Lyme borreliosis are the ticks of the genus *Ixodes* and the main species in Asia are *Ixodes persulcatus* and *I. nipponensis* [7,8]. After *B. burgdorferi* s. l. was first identified in Korea in 1993, a human Lyme borreliosis case also occurred during the same year. The incidence has increased since then [4,7]. In Korea, on average, 15.4 cases per year have been reported in the past decade, with 23 cases reported in 2019 and 18 cases reported in 2020 [9,10]. In Korea, research on *Borrelia* is currently being carried out on the pathogenicity of various wild animals, including ticks, rodents, and companion animals [3,7,11,12,13].

In Korea, *B. afzelii* and *B. garinii* were first identified in *Ixodes* ticks and wild rodents. Then, *B. bavariensis, B. tanukii, B. turdi, B. valaisiana*, and *B. yangtzensis* were identified in ticks and wild animals [7], whereas *B. afzelii* and *B. garinii* were also detected in domestic and wild animals [4,7,13]. *B. afzelii* (6/329, 1.8%) was detected in ticks collected from mammals such as horses, wild boars, native goats, and Korean water deer [4]. In a study examining the national prevalence of *Borrelia* in ticks of wild rodents in Korea, regional features of *Borrelia* spp. were observed; *B. afzelii* was found in all regions except Jeju Island, and some species were found only in the southern regions (*Borrelia valaisiana*, *B. yangtzensis*, and *B. tanukii*). Furthermore, some were found only in Jeju Island (*B. yangtzensis* and *B. tanukii*) [7]. Recently, *B. burgdorferi* sensu stricto, known to cause Lyme borreliosis in northern America, has been identified for the first time in Korea [10].

This study aimed to identify regional differences of ticks in the distribution and prevalence of *Borrelia* spp. based on tick species near a farm environment and to also identify *Borrelia* infections not only in *Ixodes* ticks, which are the major vectors of *Borrelia* spp., but also in *Haemaphysalis* and *Amblyomma* ticks.

## 2. Materials and Methods

### 2.1. Tick Collection and Species Identification

**Figure 1:**
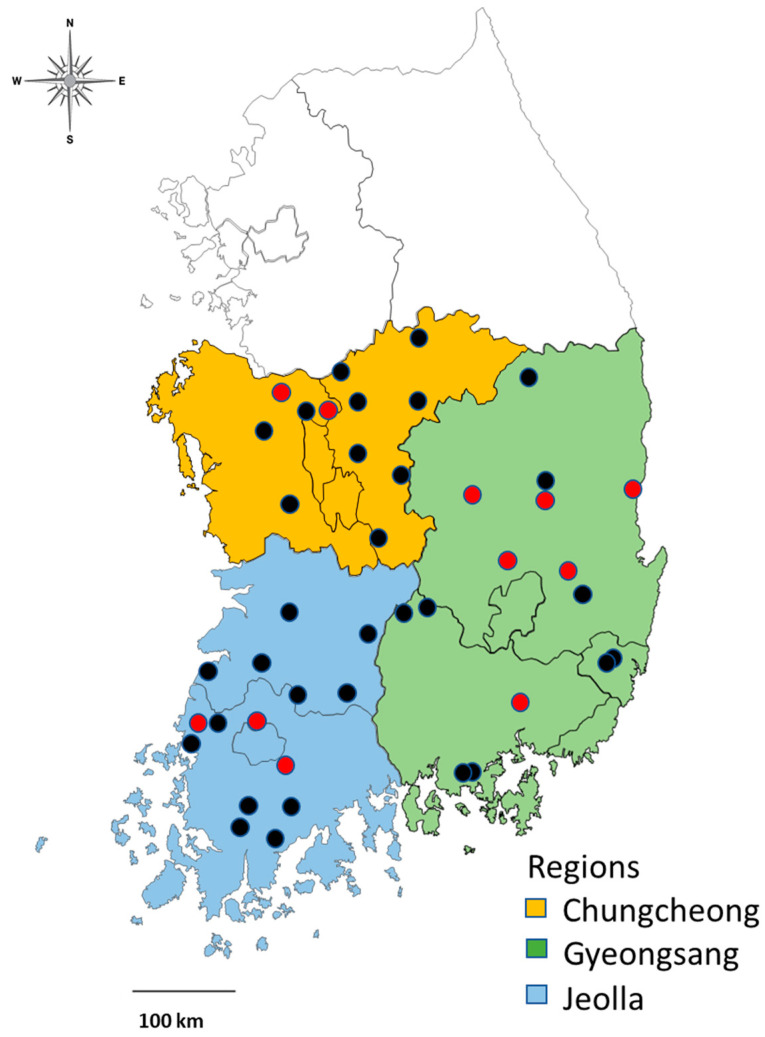
Study area, including tick collection sites in the central and southern regions of Korea (March–July 2021). Three study regions are indicated by different colors based on the administrative district boundaries (Chungcheong, yellow; Gyeongsang, green; Jeolla, blue). Tick collection sites are indicated by black circles, and sites PCR-positive to *Borrelia* by red circles.

A total of 6102 ticks were collected once a month each on an area of about 70 m^2^ grasslands and mixed forests around livestock farms (horses, deer, goats, and cattle) in three central and southern regions of Korea [Chungcheong (CC), Gyeongsang (GS), and Jeolla (JL) regions] (Figure 1) from March to July 2021. These regions were chosen due to the fact that a grant was supported to assess the tick prevalence in these areas. For tick collection, flagging and dragging were performed and the collected ticks were stored in 70% ethanol. For morphological identification, a dissecting microscope was used with reference to the previously reported standard keys [14] and species, developmental stage, and sex for adult ticks were confirmed. The sexes of adult ticks were identified based on the size of the scutum on the dorsal side.

### 2.2. DNA Extraction, PCR and Phylogenetic Analysis

For DNA extraction, the adult ticks were extracted individually; 1-13 nymphs were pooled, and up to 50 larvae were pooled. Since the ticks were pooled after identifying them by species, different species were not mixed during the DNA extraction process. However, *Haemaphysalis* larvae were pooled with *Haemaphysalis* spp. regardless of species. For DNA extraction, a commercially available DNeasy^®^ Blood & Tissue Kit (Qiagen, Hilden, Germany) was performed according to the manufacturer’s instructions. For PCR, the AccuPower HotStart PCR Premix Kit (Bioneer, Daejeon, Korea) was used as previously described for *Borrelia* spp. detection including the primers and PCR conditions for amplifying the 5S-23S rRNA intergenic spacer region (rrf-rrl) [3]. DNA templates of *B. afzelii* and *B. garinii* identified in our previous studies [3] were used as positive controls. Sterile distilled water without *Borrelia* DNA was used as the negative control. All PCR-positive products were sent to Macrogen (Daejeon, Korea) for sequence analysis and each aligned sequence was obtained using MEGA (v. 7.0; Raleigh, NC, USA) and BioEdit (v. 7.2.5; Raleigh, NC, USA). Phylogenetic trees were constructed based on the maximum likelihood method using MEGA (v. 7.0; State College, PA, USA) for the sequences obtained by BLAST search using the NCBI database (http://blast.ncbi.nlm.nih.gov/Blast.cgi; accessed on 5 September 2021) and each aligned sequence obtained in this study.

### 2.3. Statistics

Statistical analysis was performed with chi-square test using SPSS V. 26.0 (SPSS Inc., Chicago, IL, USA); *p* < 0.05 were considered significant.

## 3. Results

### 3.1. Distribution of Tick Species

A total of 6102 ticks were collected for three genera and four species (*Haemaphysalis longicornis*, *Haemaphysalis flava*, *Ixodes nipponensis*, and *Amblyomma testudinarium*) (Table 1). By region, CC 1859 (30.5%), GS 2335 (38.3%), and JL 1908 (31.3%) ticks were collected, respectively. *Haemaphysalis longicornis*, *H. flava*, and *I. nipponensis* were identified in all regions. In contrast, *Haemaphysalis* spp. larvae were identified in two regions (CC 293, 15.8%; JL 577, 30.2%), except for the GS region, and *A. testudinarium* was identified in two regions (GS 5, 0.2%; JL 108, 5.7%), except for the CC region. The most common species in all regions was *H. longicornis* with 4222 ticks (CC 1347; GS 2110; JL 765). *Haemaphysalis flava* was identified in 847 ticks (CC 201, 10.8%; GS 198, 8.5%; JL 448, 23.5%). *Ixodes nipponensis* was identified in 50 ticks (CC 18, 1.0%; GS 22, 0.9%; JL 10, 0.5%). In a total of 175 adult ticks, 80 males (1.3%) and 95 (1.6%) females were identified.

### 3.2. Prevalence of Borrelia sp.

A total of 6102 ticks (736 pools) were tested and *Borrelia*-positive ticks were detected in 20 pools (2.7% of the pools). The prevalence [minimum infection rate (MIR)] was 0.33% (Table 2). Out of 43 pools containing 50 *I. nipponensis* ticks, 17 pools (34.0% MIR) were positive. Out of 11 pools containing 113 *A. testudinarium* ticks, only one pool (0.88% MIR) was positive. Out of 526 pools containing 4222 *H. longicornis* ticks, 2 pools (0.05% MIR) were positive. Regional prevalence were 4.6%, 1.6%, and 0.9% of the GS, JL, and CC region pools, respectively. *Borrelia* infection was confirmed in all three regions of *Ixodes* ticks: 65.0% in the GS region, 20.0% in the JL region, and 15.4% in the CC region. In sexes, the prevalences were 10% and 3.2% in the adult males and females, respectively.

### 3.3. Molecular and Phylogenetic Analyses

**Figure 2:**
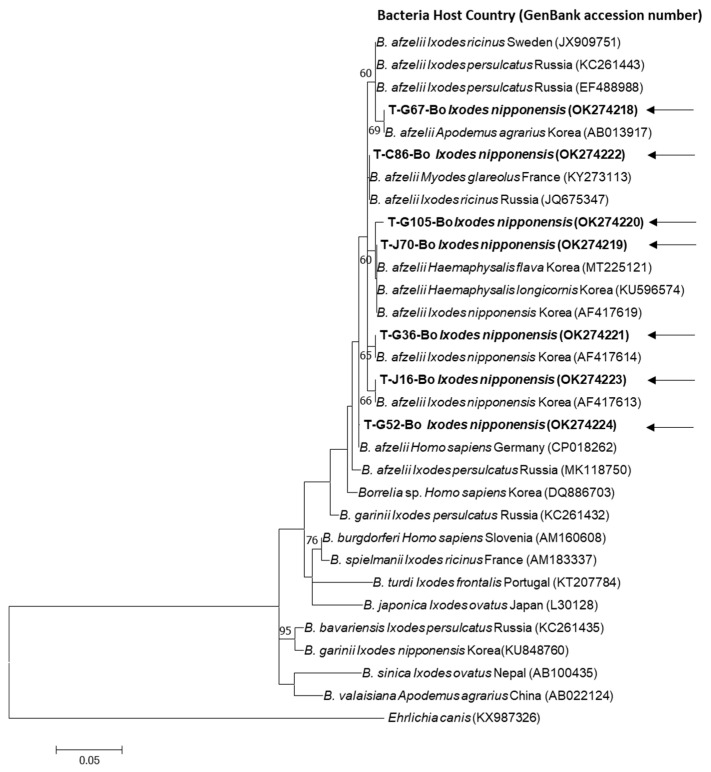
Phylogenetic analysis of *Borrelia* spp. according to the 5S–23S intergenic spacer region. The tree was constructed using the maximum likelihood method, MEGA 7.0. Sequences identified in this study are indicated by bold and black arrows.

In this study, seven representative sequences (without duplicate sequences) were identified. All seven sequences were grouped with *B. afzelii* isolates when compared to GenBank registered reference sequences. When the obtained nucleotide sequences were compared, they showed 96.7–99.5% identity. The seven sequences were submitted to GenBank (OK274218-OK274224) (Figure 2).

## 4. Discussion

In the present study, *Borrelia* spp. of 5S-23S gene sequences were detected in the tick pools (20/736, 2.7%). In Korea, *H. longicornis* was confirmed to be the dominant species [13,15]. However, *Ixodes* ticks are the main vector for *Borrelia*. Therefore, although the population was small (50 ticks), the *Borrelia* MIR was 34.00, which was higher compared with that of other ticks (*H. longicornis*: 0.05; *A. testudinarium*: 0.88). This is consistent with the results of a previous study of tick-borne pathogens in Daejeon and its surrounding areas in South Korea [8]. The values were *H. longicornis* (1.5%) and *H. flava* (0.7%), whereas *I. nipponensis* was the highest at 20.9% [8]. Of the 20 tick pools, only two were *H. longicornis* and *A. testudinarium* was detected in only one pool. To our knowledge, this is the first study in Korea to document the presence of *B. afzelii* in *A. testudinarium* ticks. In previous studies, *B. garinii* of *I. nipponensis* is found on domestic dogs and *B. afzelii* of *Haemaphysalis* ticks, which infects wild Korean water deer, were detected [3,13]. Since previous studies have shown that *H. longicornis* can carry, but cannot transmit *Borrelia* [7,16], it has been suggested that *Ixodes* ticks are the primary vehicle for *Borrelia* transmission in Korea [7]. However, this is the first identification of *Borrelia* sp. in *A. testudinarium*, suggesting that further studies are needed to determine whether *A. testudinarium* may also serve as a vector.

In the present study, most of the ticks collected were in the nymph stage (4948 ticks, 81.1%). This is related to the developmental stage of the ticks and seasonal factors, which is consistent with that of previous results [17] of tick collection from March to July. In a previous study investigating the number of ticks by developmental stage, the peak populations of nymphs, adults, and larvae were observed in April–June, June–July, and August–September, respectively [17,18]. With respect to the distribution of ticks, *H. longicornis* was found abundantly in all three regions (CC: 1374 ticks; GS: 2110 ticks; JL: 765 ticks). In Korea, *H. longicornis* was confirmed as the dominant tick species [8]. *Haemaphysalis flava* and *Haemaphysalis* spp. larvae accounted for the next highest proportion, with *Haemaphysalis* ticks accounted for 97.3% of the total. However, *Haemaphysalis* spp. larvae were not found in the GS region in this study. This may have occurred due to the fact that the size of the larva was too small when transferring the tick after flagging and dragging. *Ixodes nipponensis* and *A. testudinarium* were not prevalent (2.7%). *I. nipponensis* were identified in all regions, whereas *A. testudinarium* were not identified in the CC region. This is consistent with previous results. *A. testudinarium* was primarily found in southern regions of Korea and very rarely in other regions [8]. Finally, *A. testudinarium* was identified only at some areas south of the JL and GS regions.

We compared regional distributions between tick populations and *Borrelia* prevalence. Ticks positive with *Borrelia* were confirmed in all regions, but the GS region showed a three to five times higher prevalence than other regions (*p* = 0.016). In addition, *I. nipponensis* was detected in all three regions, and the GS region also showed a higher prevalence than other regions (*p* = 0.006). In *I. nipponensis*, the regional difference in the prevalence was significant (*p* = 0.006). However, since no historical data can be compared, it should be confirmed through continuous research.

From the results of phylogenetic analysis, we confirmed that all sequences were *B. afzelii*. In particular, the two *Borrelia*-positive sequences identified in *H. longicornis* were identical to OK274219 and OK274222 detected in *I. nipponensis* in this study, respectively. The OK274219 sequence was 100% identical to the *Borrelia* sequences of *H. longicornis* (KU596574) and *H. flava* (MT225121) previously detected in Korea. The *Borrelia* sequence of *A. testudinarium* detected in this study (identical to OK274222 sequence) was 100% identical to the sequence detected in voles in France (KY273113). The OK274224 sequence was 100% identical to the *B. afzelii* sequence (CP018262) identified in Germany.

In this study, only *B. afzelii* was detected, but previous studies confirmed *B. garinii*, which is known to be pathogenic to humans, similar to *B. afzelii*. A recent study in which *B. afzelii* and *B. garinii* was detected in wild rodent tissues showed that the *Borrelia*-positive rate was 29.8% and 25.9% for *B. afzelii* and 3.7% for *B. garinii* [10]. Another study identified *Borrelia* genospecies in various sampling areas in Korea, which were identified as *B. afzelii* (62.5%) and *B. garinii* (1.6%) [7]. As such, *B. afzelii* is more common and *B. garinii* is rare in Korea. In particular, only 100% *B. afzelii* was identified in certain areas. However, this may be due to differences in the sampling method and host, survey area, and seasonal factors, thus continuous research is needed.

In conclusion, we found that 20 out of 736 pools (6102 ticks) in central and southern Korea were positive for *Borrelia* (0.33% MIR). Among them, 17 pools were *I. nipponensis*, two pools were *H. longicornis*, and one pool was *A. testudinarium*. In *Ixodes* ticks, we found significant regional differences in *Borrelia* detection (*p* = 0.006), but this should be confirmed through further research with more ticks in a nationwide study. All positive samples were identified as *B. afzelii*. To our knowledge, this is the first study to report the presence of *B. afzelii* in *A. testudinarium*. Since *B. afzelii* is a zoonotic pathogen, it is important to gain insight into the role of *A. testudinarium* as a vector and through continuous surveillance and monitoring.

## Figures and Tables

**Table 1:** Distribution of tick species based on region and developmental stage.

Species	Stage ^1^	No. of Ticks Collected from Region			Total	*p*-Value ^2^
		Chungcheong	Gyeongsang	Jeolla		
*Haemaphysalis* spp. ^3^	Larva	293	0	577	870	-
*Haemaphysalis* *longicornis*	Nymph	1319	2059	740	4118	0.001
	Adult (M)	7	23	1	31	
	Adult (F)	21	28	24	73	
	Subtotal	1347	2110	765	4222	
*Haemaphysalis flava*	Nymph	186	179	439	804	<0.001
	Adult (M)	9	18	1	28	
	Adult (F)	6	1	8	15	
	Subtotal	201	198	448	847	
*Ixodes nipponensis*	Larva	5	0	0	5	<0.001
	Nymph	6	11	1	18	
	Adult (M)	7	11	3	21	
	Adult (F)	0	0	6	6	
	Subtotal	18	22	10	50	
*Amblyomma testudinarium*	Larva	0	0	104	104	<0.001
	Nymph	0	4	4	8	
	Adult (M)	0	0	0	0	
	Adult (F)	0	1	0	1	
	Subtotal	0	5	108	113	
Total		1859	2335	1908	6102	<0.001

^1^ M; Male, F; Female. ^2^ Significant correlation (*p* < 0.05). ^3^ *Haemaphysalis* larvae were marked as *Haemaphysalis* spp. due to difficulty in morphological identification.

**Table 2:** Prevalence of *Borrelia* in ticks collected in the central and southern regions of Korea, concerning tick species, developmental stage, and sex.

Species	Stage ^1^	Tested Tick (Pool) ^2^	No. Positive Tick Pool/Tick Pool Tested in Region ^3^			Total	MIR ^4^	*p*-Value ^5^
			CC	GS	JL			
*Haemaphysalis* spp. ^6^	L	870 (20)	0/7	0	0/13	0/20		-
*Haemaphysalis longicornis*	N	4118 (422)	0/137	1/210 (0.5%)	0/75	1/422 (0.2%)	0.02	0.688
	AM	31 (31)	0/7	0/23	0/1	0/31		
	AF	73 (73)	0/21	1/28 (3.6%)	0/24	1/73 (1.4%)	1.37	
	Subtotal	4222 (526)	0/165	2/261 (0.8%)	0/100	2/526 (0.4%)	0.05	
*Haemaphysalis flava*	N	804 (93)	0/21	0/24	0/48	0/93		-
	AM	28 (28)	0/9	0/18	0/1	0/28		
	AF	15 (15)	0/6	0/1	0/8	0/15		
	Subtotal	847 (136)	0/36	0/43	0/57	0/136		
*Ixodes* *nipponensis*	L	5 (1)	0/1	0	0	0/1		0.006
	N	18 (15)	1/5 (20.0%)	6/9 (66.7%)	0/1	7/15 (46.7%)	38.89	
	AM	21 (21)	1/7 (14.3%)	7/11 (63.6%)	0/3	8/21 (38.1%)	38.10	
	AF	6 (6)	0	0	2/6 (33.3%)	2/6 (33.3%)	33.33	
	Subtotal	50 (43)	2/13 (15.4%)	13/20 (65.0%)	2/10 (20.0%)	17/43 (39.5%)	34.00	
*Amblyomma testudinarium*	L	104 (4)	0	0	0/4	0/4		1.000
	N	8 (6)	0	0/2	1/4 (25.0%)	1/6 (16.7%)	12.50	
	AM	0	0	0	0	0		
	AF	1 (1)	0	0/1	0	0/1		
	Subtotal	113 (11)	0	0/3	1/8 (12.5%)	1/11 (9.1%)	0.88	
Total		6102 (736)	2/221 (0.9%)	15/327 (4.6%)	3/188 (1.6%)	20/736 (2.7%)	0.33	0.016

^1^ L; Larva, N; Nymph, AM; Adult Male, AF; Adult Female. ^2^ Adult ticks were not pooled and each individual was tested. ^3^ CC; Chungcheong, GS; Gyeongsang, JL; Jeolla. ^4^ Estimated as Minimum Infection Rate (MIR). Calculated as number of positive pools/total number of ticks tested ×100. ^5^ Significant (*p* < 0.05) correlation with infection. ^6^ *Haemaphysalis* larvae were marked as *Haemaphysalis* spp. due to difficult morphological identification.

## Data Availability

Data supporting the conclusions of this article are included within the article. The newly generated sequences were submitted to the GenBank database under the accession numbers OK274218-OK274224. The datasets used and/or analyzed during the present study are available from the corresponding author upon reasonable request.
